# Effectiveness and cost of an incentive-based intervention on food safety and income in “dibiteries” in Dakar, Senegal

**DOI:** 10.1186/s12889-022-12812-x

**Published:** 2022-03-11

**Authors:** Malik Orou Seko, Walter Ossebi, Clarisse A. Houngbedji, Katharina Kreppel, Daouda Dao, Bassirou Bonfoh

**Affiliations:** 1 Ecole Inter-Etats des Sciences et Médecine Vétérinaires, Dakar, BP 5077 Fann-Dakar, Sénégal; 2 Centre d’Entomologie Médicale et Vétérinaire, Université Alassane Ouattara, Bouaké, Côte d’Ivoire; 3 Centre Suisse de Recherches Scientifiques en Côte d’Ivoire, Abidjan, 01BP 1003 Abidjan 01, Côte d’Ivoire; 4 Nelson Mandela African Institution of Science and Technology, P.O. Box 447, Arusha, Tanzania; 5 Institute of Tropical Medicine, Antwerp, Belgium; 6 Université Félix Houphouët-Boigny, Abidjan, 01 BP 34 Abidjan 01, Côte d’Ivoire; 7 Swiss Tropical and Public Health Institute, P.O. Box 4002, Basel, Switzerland; 8 University of Basel, P. O. Box 4001, Basel, Switzerland

**Keywords:** Meat, Dibiterie, Intervention, Hygiene training, Cost, Effectiveness, Senegal

## Abstract

**Background:**

Rapid urbanisation in Sub-Saharan African cities such as Dakar, Senegal, leads to proliferation of informal braised meat restaurants known as “dibiteries”. Dibiteries do not often comply with minimal hygiene and food safety standards. The primary objective of this study was to assess the effectiveness and cost of a good hygiene practice intervention, identify factors that incentivize hygiene improvement and how that impacts on dibiteries’ income.

**Methods:**

A randomized controlled trial was carried out in Dakar dibiteries. The 120 random samples of braised meat were collected in three phases: (i) one-month pre-intervention, (ii) 2 months post-intervention, (iii) 10 months post-intervention. The trial comprised four groups of 10 dibiteries each: (*a*) (control) received no intervention, (*b*) a standardized training module, (*c*) a hygiene kit, (*d*) a training module and hygiene kit. Laboratory analysis of samples determined the total aerobic mesophilic flora (TAMF), thermotolerant coliforms (TC) and *Staphylococcus aureus* (SA). A questionnaire-based survey and focus group discussion were used to identify pre-intervention hygiene practices, and socioeconomic determinants of hygiene management in dibiteries post-intervention, respectively.

**Results:**

Samples were found to be contaminated with TAMF, TC and SA. In phase 1, 27 and 13% of the samples contained TC and SA, respectively. In phase 2, no significant improvement of contamination rates was seen. In phase 3, microbiological quality of samples was significantly improved, with only 11.5% showing contamination with any of the bacterial species analysed (*p* < 0.1). Compared to the control group, only samples from dibiteries in group (*b*) had significantly reduced bacterial load in phase 3. The cost of intervention and hygiene improvement was estimated at 67 FCFA ($ 0.12) and 41 FCFA ($ 0.07) / day respectively and did not significantly impact on dibiterie profitability. Incentives to sustainably implement good hygiene practices were mainly linked to access to secure long-term workspaces.

**Conclusion:**

This intervention may have worked, but globally the results are mixed and not quite significant. However, continuous training in good hygiene practice and access to secure and sustainable infrastructure for dibiterie restaurants are the incentives necessary to achieve sustainable investments and behavioural change. We recommend further intervention refinement and testing other factors for promoting the adoption of good hygiene practices in the dibiteries in relation to consumers health risk.

## Background

Food safety is an essential component of public health due to the considerable burden of foodborne diseases, especially in Africa. According to Word Health Organisation (WHO) estimations, more than 91 million people fell ill due to consuming unsafe foods in the African region in 2015. This resulted in 137,000 deaths, representing one-third of the global mortality from foodborne diseases [1].

In sub-Saharan Africa, this major public health problem can be related to the sale of a wide variety of animal source products in the informal food market system. More than 80% of food is sold in informal markets and about 80% of the population of towns, including schoolboys, students, employees, unemployed, street children and traders, eat in the informal food market system easily outside the home and at low cost [2–4]. Food safety is compromised on many levels of the informal market including wet markets, transport and storage, and production and sale (ready-to-eat food restaurants).

At the wet market level, a large portion of the animal source products such as meat, milk, eggs and fish are sold in traditional local markets lacking modern infrastructure including access to clean running water and electricity, and escape any food safety regulations and food control. In addition, individuals trading in these informal markets often have no training in good hygiene and manufacturing practices when handling food [4]. At the transport and storage level, the unavailability of appropriate transport means, and storage and refrigeration facilities lead to high losses or to practices that threaten food safety and the health of consumers [4]. Indeed, the products intended for the market are often transported in unsuitable means of transport, causing a break in the cold chain and consequently environmental cross contamination, and the multiplication of pathogenic microorganisms. Problems at the level of production and sale mainly concern ready-to-eat food restaurants preparing and selling foods containing animal products in precarious settings and using unhygienic practices. Most of the time, these foods originate from restaurants operating in the informal sector, where traditional preparation methods of agri-food products are predominant. These traditional methods are characterised by manual processing of foods and, in some cases, laborious and unhygienic operating procedures [2].

Undoubtedly, pathogens such as toxin-producing *Escherichia coli* (*E. coli*) and *Salmonella* spp.*,* which can induce foodborne diseases in consumers, are found in informal markets. Several studies have measured the prevalence of foodborne pathogens in informal markets. According to the study by Adesokan et al. [5], 75.5, 65.2, 61.6, and 46.9% of meat samples collected from informal market in Nigeria were positive for *Staphylococcus aureus*, *Listeria monocytogenes, Salmonella spp*, and *E. coli*, respectively. In addition, these authors obtained significant higher prevalence from the informal markets for *Staphylococcus aureus* (OR = 9.43; 95%CI: 0.05–0.24), *Listeria monocytogenes* (OR = 9.35; 95%CI: 0.06–0.21), *Salmonella spp* (OR = 10.00; 95%CI: 0.05–0.19) and *E. coli* (OR = 12.99; 95%CI: 0.04–0.15) than the formal markets [5]. Thus, the control or improvement of the hygiene deficits observed in informal food markets, through cost-effective interventions, would generate considerable economic gain in term of income for informal processors of animal derived foods, in addition to health and economic benefits for consumers [4, 6].

In Senegal, rapid urbanisation of cities such as Dakar leads to the proliferation of informal ready-to-eat food restaurants, so-called ‘dibiteries’, preparing and selling braised sheep, goat, poultry meat. However, in these ready-to-eat restaurants, there are observed risky behaviours and practices that have the potential to contaminate food with pathogens. These include the use of iced-water (which often come from non-potable water) for the conservation of the meat during power outages, hanging of the meat in the open air, money handling practices of sellers, and use of recycled cement bags for meat packaging. Indeed, a qualitative risk assessment of food safety in the small ruminant value chain showed that around 50% of dibiterie restaurants sold braised meat contaminated with faecal coliforms. *E. coli* and total aerobic mesophilic flora (TAMF) contributed 45% each to the observed contaminations [7]. In addition, these risky behaviours coupled with the lack of knowledge of good hygiene practices by dibiterie staff, were associated with a 51% probability of having a quite high to very high microbial contamination in the dibiterie’s meat [7]. This means that dibiterie meat samples have a one out of two chance of having quite high or very high contamination. These hazards observed have not yet been studied on how it translates into health risk for consumers. However, the dibiterie business is very profitable and generates net profits of around USD 525.5 per month per tenant [8]. Therefore, dibiteries seem to operate under the principle of profit maximization to the detriment of quality and safety.

Unfortunately, so far, no hygiene interventions have been implemented in the dibiteries. In light of this, our study sought to evaluate whether intervention for improving the quality of food preparation practices in the dibiteries affects their profitability. Specifically, this involved (i) assessing the attitudes and practices of the dibiterie tenants toward hygiene prior to intervention; (ii) evaluating the effectiveness of intervention on the microbiological quality of dibiterie meat; (iii) estimating the cost of the hygiene improvement and intervention package, and its impact on the economic outcomes of dibiteries; and finally (iv) identifying the factors that incentivize the enhancement of the hygiene practices and meat quality in dibiteries in Dakar.

## Methods

### Study design and study area

**Fig. 1:**
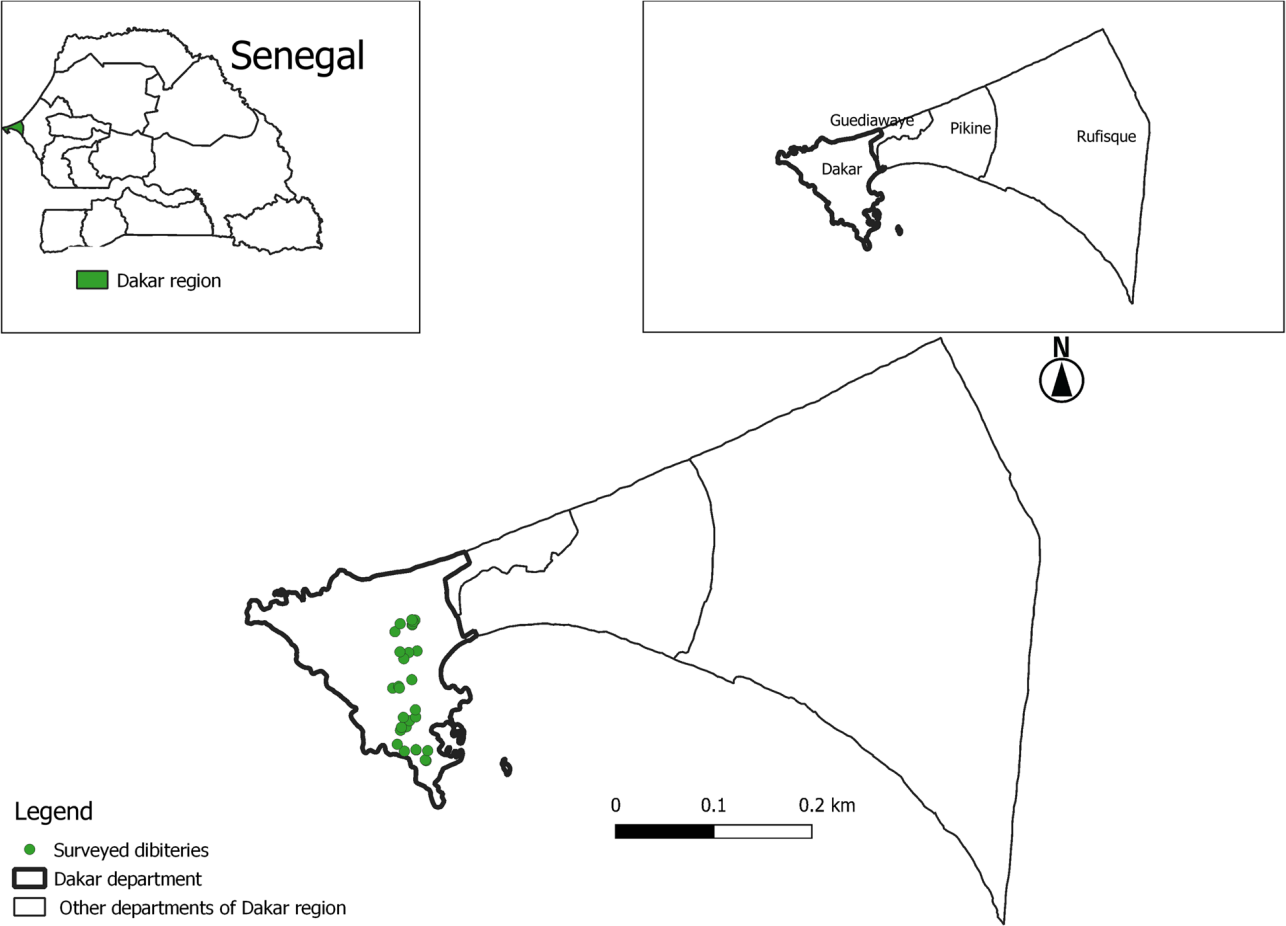
Geographical location of the dibiteries chosen for intervention in Dakar city, Senegal

A randomized controlled trial study was carried out in dibiteries of the city of Dakar in Senegal between May 2015 and March 2018 (Fig. 1) by following the relevant Consolidated Standards of Reporting Trials (CONSORT) statement and guidelines [10]. This study included three phases, a questionnaire-based survey and a focus group discussion on the management of hygiene practices and economic performance of the dibiteries; and laboratory-based microbiological analyses of braised meat samples from these dibiteries.

The city of Dakar is the capital of Senegal in West Africa. Dakar is an Atlantic port city located on the Cap-Vert peninsula. Monthly temperatures range between 25 °C and 30 °C with an annual rainfall of 469 mm (https://www.en.climate-data.org). With a population of 1 million people, the city is characterised by a high demand for meat products in Senegal. The ever-increasing demand for meat products associated with a demographic surge has thus led to an increased concentration of dibiterie establishments in Dakar.

### Sampling and intervention scenario

The sample base for the present study consists of 40 randomly selected dibiteries used by a previous study by Yougbare [7] on the qualitative assessment of the risks of microbial contamination of small ruminant meat in slaughterhouses and dibiteries of Dakar. This cross-sectional study allowed to make an initial assessment of the hygiene and microbiological quality of the meat, the cleanliness of the premises of the dibiterie.

The eligibility criteria for inclusion in the study were that the dibiteries must come from the study by Yougbare [7]; give their consent to be included in the intervention by sign the informed consent form; and be operational during all the duration of the intervention.

The intervention scenario included two factors: (i) sensitization and training regarding good hygiene and production practices and (ii) distribution of a hygiene kit (hygiene materials and consumables). The 40 dibiteries in which the first evaluation of the hygiene and microbiological quality of the meat was carried out were randomly split, but in a reasoned way, into four groups of 10. Group (*a*), the control group, received no intervention; group (*b*) received training; group (*c*) received a hygiene kit and group (*d*) received training and a hygiene kit. The intervention groups (*b*, *c,* and *d*) included two participants per dibiterie, one each from the employer / employee level (owner-manager and simple employee, or manager-employee and simple employee). Thus, two people per dibiterie participated in the training workshop and / or received the hygiene kits. Each dibiterie volunteered 2 employees to participate in the intervention by giving prior informed written consent. Concerning the sequence of the intervention, a training workshop was given to the groups *b* and *d* simultaneously, followed by the distribution of the hygiene kits to the groups *c* et *d* on the same day of the training workshop. However, the different groups of the intervention were distributed and the order of the intervention methods were designed to reduce the likelihood of introducing bias among the intervention groups and to better appreciate the effectiveness of the messages promoted. Indeed, the groups of the intervention were distributed according to the provenance areas of the participants. The groups have been distanced from each other so that they cannot interact between them. The training workshop delivered awareness messaging based on good hygiene practices on the premises, personal and clothing hygiene, material and equipment hygiene, and packaging of the finished products. The key messages were elaborated based on the risky practices related to hygiene identified by Yougbare [7]. The training workshop was given in face-to-face by PowerPoint presentation at the Ecole Inter-Etats des Sciences et Médecine Vétérinaires of Dakar by the supervisors (last co-author). For each mode of intervention, a presentation of the promoted messages was given and ended with a general discussion managed by the first author. The duration of the training was 3 h (09 AM to 12 PM) while the hygiene kits were distributed in 4 h (from 2 PM to 6 PM) within the dibiteries. Hygiene kits were delivered to dibiteries by the fist author. Overall discussions included issues ranging from handwashing and disinfection, consequences of hanging meat in the open-air, treating and protecting skin infections and injuries, to the frequency of cleaning and disinfection of equipment, etc. Cleaning and disinfection, however, was discussed in more detail than the other issues. The messaging provided on this topic included three main steps such as the procedures (i) before cleaning, (ii) during cleaning, and (iii) during disinfection. The nature of the key messages and the cleaning and disinfection procedures are presented in detail in Traoré et al. [8]. The hygiene kit distributed to each dibiterie in group (*c*) and (*d*) consisted of a scrubbing brush, bucket, mop, trash can, white coat, cap, apron, liquid soap (5 L), bleach (5 L), and paper towels, which is equivalent to a total value of 37,900 FCFA (USD 68) per dibiterie.

### Monitoring of the intervention and data collection from the dibiteries

Monitoring of the intervention was performed through the collection of information and meat samples from the 40 dibiteries in three distinct phases. The information was collected through a structured questionnaire. In addition to the sociodemographic characteristics of the dibiterie tenants, the questionnaire was focused on the attitudes and practices toward hygiene and their different economic costs and daily production revenues (cost of equipment, production materials, quantities and prices of sale of products).

At each phase, only one sample of braised meat was purchased (1 kg) per dibiterie. The samples consisted of braised meat freshly cooked in a traditional oven, together with ingredients such as onions, peppers, *Kankan* (cocktail of condiments and spices consisting of peanut oilcake, chilli powder, pepper, broth, salt and garlic), and mustard according to the specific cooking method of the dibiteries [8, 9]. The core cooking the oven temperature and the cooking time of the braised meat were not measured. In general, the dibiterie tenants estimated the proper cooking time and temperature; according to them, well cooked meat has no more blood [8]. This guideline was used to determine the cooking level and to choose the samples of well-cooked braised meat. In total, 120 samples of the mixture were collected and about 100 g were sent to the laboratory of Hygiène et Industries des Denrées Alimentaire d’Origine Animales (HIDAOA) at the Ecole Inter-Etats des Sciences et Médecine Vétérinaires (EISMV) of Dakar for microbiological analyses. They were immediately treated according to the protocol defined by French (AFNOR) and ISO standards for the detection and enumeration of three (03) indicators of microbiological contamination, in particular Thermotolerant Coliforms (TC; ISO 4832), Total Aerobic Mesophilic Flora (TAMF; NF EN ISO 4833) and *Staphylococcus aureus* (SA; NF EN ISO 6888). These indicators were chosen on the basis of the germs identified in the study carried out by Yougbare [7] on the qualitative assessment of the risks of microbial contamination of small ruminant meats in slaughterhouses and dibiteries in Dakar.

Phase 1 was carried out 1 month (May 2015) prior to the implementation of the intervention package (training and hygiene kit distribution in June 2015). For this baseline survey samples of approximately 100 g of dibiterie meat were taken from each dibiterie and a structured questionnaire was administered to one of the two employees who agreed to take part in the study in each of the 40 chosen dibiteries. The questionnaire was administered in person in the local Wolof language.

Phase 2 and 3 were carried out 2 months (August 2015) and 10 months (April 2016) after the distribution of the intervention package respectively. During each of these phases, samples of about 100 g of dibiterie meat were also collected from the 40 dibiteries.

**Fig. 2:**
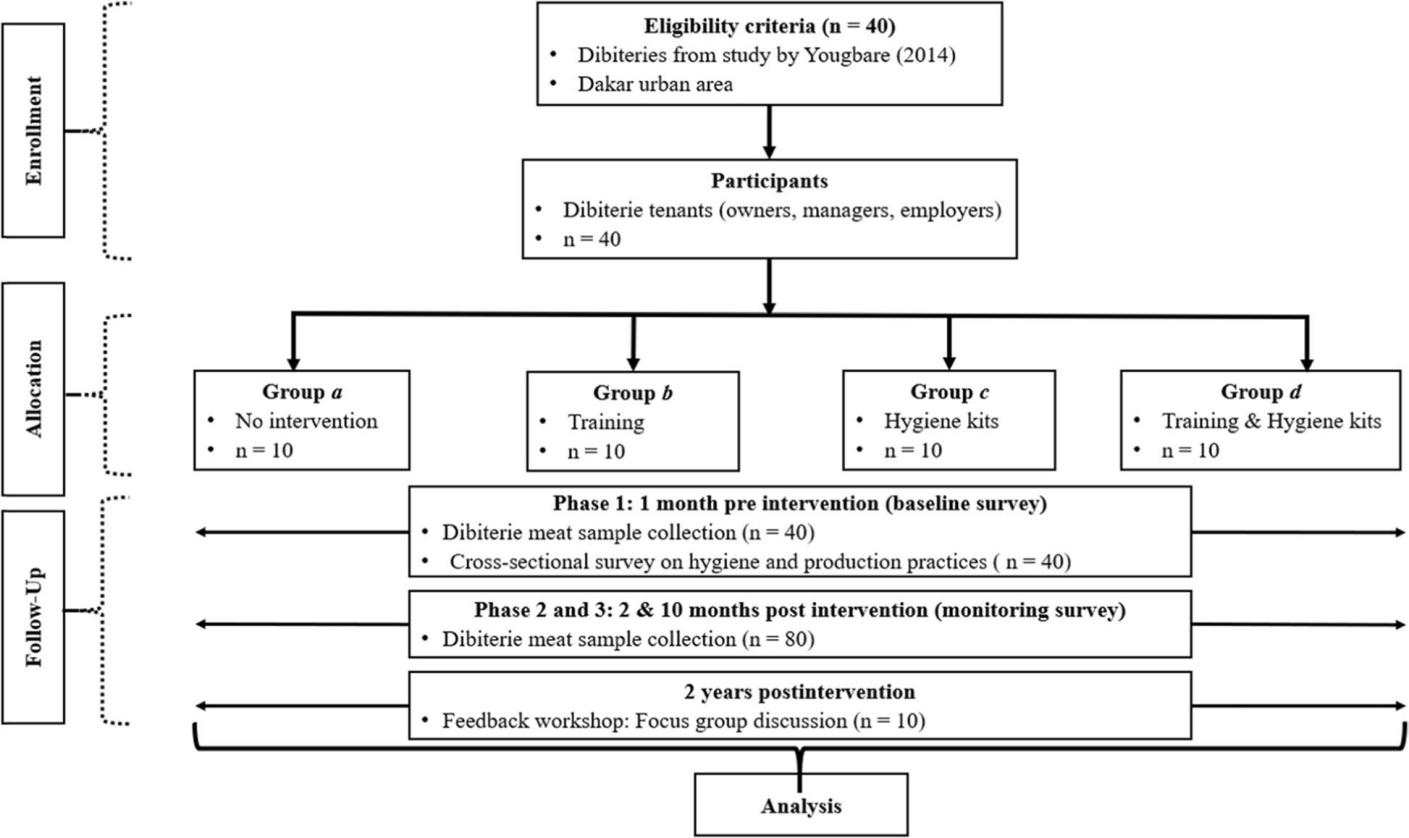
Diagram of the intervention scenario

A feedback workshop of the study was organized two-years post-intervention with dibiterie tenants, hygiene service officers, and livestock directorate officers to establish a dialog and co-design an incentivised and cost-effective intervention. During this workshop, a focus group discussion (FGD) was carried out using an interview guide with 10 dibiterie tenants at the Ecole Inter-Etats des Sciences et Médecine Vétérinaires (EISMV) of Dakar. The FGD was done in French and translate in Wolof, a local language. The 10 dibiterie tenants were randomly selected from the 4 intervention groups; two people each were chosen from groups (*b*) and (*c*) and three people each from groups (*a*) and (*d*). This distribution was made in order to better perceive the impact of the intervention on the group that received the entire hygiene package compared to the control group. The interview guide allowed collecting information on hygiene management and its implication on product quality as well as socio-economic determinants that could impact the good hygiene practices in dibiteries. The FDG was about 50 min long. Figure 2 shows the flow chart of the intervention scenario.

### Estimation of the costs of hygiene improvement and the intervention package

The costs of hygiene improvement and the hygiene kit were estimated by evaluating the costs of materials and consumables and the depreciation of the equipment used with the usual hygiene practices in all the dibiteries. They were estimated respectively based on the Eqs. (1) and (2) of Bonfoh et al. [11].

1 $$\left\{\begin{array}{c} CHI={CH}_f-{CH}_i\\ {} Knowing\ that:\\ {}{CH}_i={CE}_i+{CC}_i\\ {}{CH}_f={CE}_f+{CC}_f\end{array}\right.$$

With: CHI = Cost of Hygiene Improvement; CH = Cost of Hygiene Practices; CE = Cost of Equipment; CC = Cost of Consumables; *i* = without intervention; *f* = with intervention

2 $$\left\{\begin{array}{c} CHK=\left[{\left( IE+ RF\right)}_f-{\left( IE+ RF\right)}_i\right]\ast \frac{1}{D_x}\\ {} RF= OC-\left( Lo+ FA\right)\end{array}\right.$$

With: CHK = daily cost of hygiene kit (FCFA / day); IE = Investment in the equipment and materials (FCFA, USD); RF = Revolving funds (FCFA); Dx = the life period or use of the equipment invested (day); OC = Own capital (FCFA); Lo = Loans (FCFA); FA = Fixed assets (FCFA); *i* = before intervention; *f* = after intervention.

The cost of training per dibiterie was estimated by listing all expenses incurred during the workshop, including travel expenses, room rental, catering and trainer fees.

Finally, the cost of the intervention package is the sum of the daily costs of the hygiene kit and the training.

### Data statistical analysis

The data collected at the end of the various investigations were recorded in Excel and were subsequently transferred into the R commander software (version 3.5.1) for statistical analyses. The results of the microbiological quality of the dibiterie meat were classified into ‘satisfactory’, ‘acceptable’ and ‘not satisfactory’ according to the criteria of the laboratory of HIDAOA at EISMV. These criteria allow determining the quality of foodstuffs according to standards DGAL/SDHA/N2001–8090 for TAMF and SA and CE 1441/2007 for CT. The interpretation of the results was derived from a 3-class plan and was carried out as follows: (i) satisfactory = below the standard or up to 3 times the standard; (ii) acceptable = between 3 to 10 times the standard; (iii) not satisfactory = greater than the standard or greater than 10 times the standard [12]. Thus, to consider the degree of contamination of a sample ‘satisfactory’ regarding the three bacterial counts, all the results had to be satisfactory. Samples were classified as ‘not satisfactory’ if at least one of the results of the sample was not satisfactory. To be classified ‘acceptable’ at least one result had to be acceptable and the sum of the others satisfactory.

Furthermore, given the non-Gaussian distribution of the data on the bacterial species (TAMF, TC, SA, Total Load), the results were presented in log_10_ CFU / g of braised meats, tested to have a normal distribution. Generalised Linear Model (GLM) allowed assessing the effectiveness of the intervention groups (*b*, *c* and *d*) on total bacterial load and by species (TAMF, TC, SA) of the braised meat samples at phases 2 and 3, compared to the control group (*a*). The response variables were the bacterial species and total bacterial load of the samples. The explanatory variables were the three intervention groups. An intervention (group) was judged effective when there was a significant difference between the control group and an intervention group with regards to bacterial loads (*p* ≤ 0.05). The *t*-test was used at the significance level of 5% to compare the average gross margins generated by the dibiterie with and without the hygiene kit.

The discussion during the FGD were recorded using a digital voice recorder, and transcribed in French in Microsoft Word. Transcripts were analysed without software. Thematic content analysis was used for the interpretation of the data. The data was classified into themes and sub-themes and presented as narratives supported by verbatim quotations from the FDG.

Moreover, all methods were performed in accordance with the relevant guidelines and regulations.

## Results

In total, all of the 40 dibiteries selected agreed to participate in the study and no drop-outs were observed during the implementation.

### Sociodemographic characteristics of dibiterie tenants receiving the intervention

The majority of the dibiteries involved in the intervention (68%) had no previous hygiene training for their staff, nor did they have a business licence (90%). Most of the dibiterie tenants were between 30 and 49 years old (63%) and 65% had no formal education. None of the dibiterie tenants owned the premises where they operated. There was no significant difference between the different intervention groups according to the descriptive characteristics of the dibiterie tenants (Table 1).

**Table 1:** Descriptive statistics of dibiterie tenants (*n* = 40)

Characteristics of the study population	Modalities	Frequency (%)	Control (%)	Training (%)	Hygiene kit (%)	Training, Hygiene Kits (%)	*p*-value
Age	15–29	6 (15)	1 (17)	2 (33)	1 (17)	2 (33)	0.369
	30–49	25 (63)	6 (24)	8 (32)	7 (28)	4 (16)	
	≥ 50	9 (22)	3 (33.3)	0 (0)	2 (22.2)	4 (44.4)	
Level of formal education	None	26 (65)	7 (27)	7 (27)	7 (27)	5 (19)	0.942
	Primary	10 (25)	2 (20)	2 (20)	3 (30)	4 (30)	
	Secondary	3 (8)	1 (33.3)	1 (33.3)	0 (0)	1 (33.3)	
	University	1 (2)	0 (0)	0 (0)	0 (0)	1 (100)	
Ownership status of the dibiterie premise	Not owner	40 (100)	10 (25)	10 (25)	10 (25)	10 (25)	N/A
	Owner	0 (0)	0 (0)	0 (0)	0 (0)	0 (0)	
Authorisation for installation	Yes	4 (10)	0 (0)	1 (25)	3 (75)	0 (0)	0.167
	No	36 (90)	10 (28)	9 (25)	7 (19)	10 (28)	
Previous hygiene training for staff	Yes	13 (32)	3 (23)	2 (15)	4 (31)	4 (31)	0.879
	No	27 (68)	7 (26)	8 (30)	6 (22)	6 (22)	

### Attitudes and practices of dibiterie tenants toward hygiene prior to the intervention

The attitudes and practices of dibiterie tenants toward hygiene prior to the intervention are presented in Table 2. The majority of dibiterie tenants (70%) did not have a medical certificate and did not stop work when they fell ill. Medical care was sought at a rate of twice per year for 20% of dibiterie tenants and three times a year for 10% of tenants.

**Table 2:** Attitudes and practices toward hygiene in dibiteries before the intervention (*n* = 40)

Category	Item	Yes (%)	No (%)
State of the staff health	Medical examination / Medical certificate	12 (30)	28 (70)
Hygiene around the raw meat	Meat transport mode: Non-refrigerated vehicles (taxi, car)	36 (90)	4 (10)
	Meat transport mode: Motorcycle	4 (10)	36 (90)
	Exposure of the meat in a covered environment	9 (22)	31 (78)
	Presence of other foods in the refrigerator	10 (25)	30 (75)
	Pest control measures	16 (40)	24 (60)
Material and equipment hygiene	Presence of hot water posts	0 (0)	40 (100)
	Cutting board: Wood	38 (95)	2 (5)
	Cutting board: Cardboard	2 (5)	38 (95)
	Presence of dirt on the cutting board	33 (83)	7 (17)
	Presence of dirt on the worktop	28 (70)	12 (30)
	Presence of toilet	8 (20)	32 (80)
	Presence of washbasin / hand washing device	37 (93)	3 (7)
	Cleanliness of non-disposable towels / handkerchiefs	11 (27)	29 (73)
	Presence of disposable hand towel	0 (0)	40 (100)
Hand and clothing hygiene	Butcher/cook also acts as the cashier	39 (98)	1 (2)
	Injury contraction during meat handling	21 (53)	19 (47)
	Hand washing after each interruption	6 (15)	34 (85)
	Wearing watches / jewellery	34 (85)	6 (15)
	Wearing regulation work clothes	1 (2)	39 (98)
	Cleanliness of work clothes (regulation or not)	34 (85)	6 (15)
Braised meat packaging material	Recycled cement bag paper	17 (42)	23 (58)
	Recycled milk bag paper	9 (22)	31 (78)
	Recycled cement bag paper and milk bag paper	5 (22)	35 (88)
	Recycled cement bag paper and aluminium paper	4 (10)	36 (90)
	Recycled milk bag paper and aluminium paper	4 (10)	36 (90)
	Butcher’s paper and aluminium paper	1 (2)	39 (98)

Meat was transported from slaughterhouses to dibiteries without cold chain (90% by taxi and cars; 10% by motorcycles). In addition, only 26% of dibiteries did not hang their meat in the open air, 60% did not carry out any pest control, and 75% of those using a refrigerator keep other foods (cooked rice, fruit, drinks) with the meat in the same refrigerator.

Regarding the hygiene of the materials and equipment, the meat cutting support was made of wood (95%) with a deposit of fat, dust on the cutting board and the work surface in 83 and 70% of cases respectively. No hot water was used in the dibiteries where 93% had a washbasin or a hand washing device and 80% did not have a toilet. Among the dibiteries which had toilets (20%), 5/8 toilets were still functional. Non-disposable handkerchiefs / towels were not clean (73%). These different materials were rarely cleaned and disinfected (1–2 times a week). In 98% of dibiteries, the salesperson acts as the cashier at the same time and 85% did not wash their hands after each interruption when handling of cash. Additionally, 53% of salespersons reported having frequent injuries to their hands while handling the meat and 85% wore jewellery / watches when handling meat. Work clothes (regulation or not) were mostly clean (85%) with only 3% of dibiteries having regulation work clothes (white coat, cap, apron). The packaging material for the dibiteries’ meat was mostly recycled cement bags (43%) and milk packaging paper (23%). Only 3% used butcher’s paper combined with aluminium foil for packaging braised meat.

### Microbiological quality of dibiterie meat and effectiveness of intervention

#### Microbiological quality of the dibiterie meat prior to and after the intervention

At phase 1, microbiological analysis of the samples showed that the dibiterie meat was contaminated with TAMF, TC, and SA. The total bacterial load averaged 4.9 log_10_ CFU / g with 30% of samples of non-satisfactory microbiological quality. However, the number of TAMF was 4.7 log_10_ CFU / g and the load of TC and SA were of 2.2 log_10_ CFU / g and 2.3 log_10_ CFU / g respectively. At this phase, 27 and 13% of the samples were of non-satisfactory quality for TC and SA.

Furthermore, at phase 2, the quality of the dibiterie meat samples had undergone a non-significant decrease in proportions of sample of non-satisfactory quality for each microbiological indicator (Table 3). On the other hand, at phase 3, a significant reduction of the proportion of samples of non-satisfactory quality was noted for the TC and for the total bacterial load (*p* < 0.1). Between phase 1 and 3, the microbiological quality of the samples improved significantly by 18.5 percentage points for the whole bacterial species (OR = 3.3; *p* < 0.1) and by 19 points of percentage for TC (OR = 4.4; *p* < 0.1).

**Table 3:** Microbiological quality of dibiterie meat samples in Dakar (*n* = 30)

Bacterial species	Before intervention		After intervention							
	Phase 1 (1 month before, *n* = 30)		Phase 2 (2 months after, *n* = 30)				Phase 3 (10 months after, *n* = 30)			
	Average (log_10_ CFU/g)	Non-satisfactory (%)	Average (log_10_ CFU/g)	Non-satisfactory (%)	OR	*p*	Average (log_10_ CFU/g)	Non-satisfactory (%)	OR	*p*
Mesophilic flora (TAMF)	4.7 ± 0.9	0	4.9 ± 0.8	0	NC	–	4.6 ± 1.4	3.8	NC	–
Thermotolerant coliforms (TC)	2.2 ± 1.7	26.7	1.5 ± 1.4	20	1.5	0.52	1.4 ± 1.2	7.7	4.4	0.06*
*Staphylococcus aureus* (SA)	2.3 ± 0.8	13.3	2.3 ± 0.7	10	1.4	0.69	2.1 ± 0.5	3.8	3.8	0.21
Samples (all bacterial species)	4.8 ± 0.9	30	4.9 ± 0.8	20	1.7	0,37	4.6 ± 1.3	11.5	3.3	0.09*

*significant at *p* < 0.1; *NC* not calculated, *OR* odd ratio

Globally, the intervention results were not quite significant regarding the improvement of the microbial quality of the dibiterie meat on the whole phases of the trial.

#### Effectiveness of intervention groups on reducing microbial contamination of dibiterie meat

The results of the logistic regression (Table 4) show the level of effectiveness of the different intervention groups on reducing the total bacterial load and by bacterial species found in the dibiterie meat. Group b (training) was found to significantly reduce (*p* < 0.05) the load of SA at phase 2, as well as the total bacterial load, and the load of TAMF and TC (*p* < 0.05) at phase 3. Regarding group c (hygiene kit), there is a non-significant reduction of the dibiterie meat contamination by TC and SA in phase 2, as well as by TAMF, TC and the whole range of bacterial species in phase 3. Group d (training + hygiene kit) was significantly effective on reducing the contamination of dibiterie meat by SA at phase 2 and by TC at phase 3 (*p* < 0.1).

**Table 4:** Effectiveness of intervention groups on the total bacterial load and the loads by bacterial species of dibiterie meat in Dakar

Groups	Bacterial species	Phase 2				Phase 3			
		Coefficient	mean estimate	95%CI	***p***-value	Coefficient	mean estimate	95%CI	***p***-value
Group (*b*)	TAMF	−1.34	0.26	[−3.05; 0.35]	0.130	−3.44	0.03	[−6.36; −0.54]	0.027**
	TC	−1.82	0.16	[−5.07; 1.41]	0.276	−3.31	0.04	[−6.34; − 0.29]	0.039**
	SA	−2.00	0.13	[−3.73; − 0.28]	0.029**	0.60e-17	1.00	[−0.96; 0.96]	1.000
	Samples (all bacterial species)	−1.36	2.55	[−3.06; 0.32]	0.122	−3.44	0.03	[−6.33; −0.57]	0.025**
Group (*c*)	TAMF	1.06	2.89	[−0.64; 2.76]	0.230	−0.37	0.69	[−3.28; 2.54]	0.803
	TC	−0.03	0.97	[−3.27; 3.21]	0.986	−0.69	0.50	[−3.72; 2.34]	0.658
	SA	−0.33	0.71	[−2.07; 1.39]	0.704	0.67	1.97	[−0.28; 1.63]	0.174
	Samples (all bacterial species)	1.03	2.80	[−0.66; 2.72]	0.240	−0.37	0.68	[−3.26; 2.49]	0.798
Group (*d*)	TAMF	−0.56	0.57	[−2.27; 1.13]	0.517	−1.70	0.18	[−4.69; 1.29]	0.275
	TC	−0.06	0.93	[−3.31; 3.17]	0.968	−3.09	0.05	[−6.22; 0.03]	0.061
	SA	−1.64	0.19	[−3.37; 0.08]	0.071	0.26e-17	1.00	[−0.99; 0.99]	1.000
	Samples (all bacterial species)	−0.58	0.56	[−2.28; 1.10]	0.501	−1.73	0.18	[−4.70; 1.23]	0.259

** significant at *p* < 0.05; *CI* confidence interval

Overall, the effectiveness of the intervention had “unclear” or “equivocal”, the results of the intervention groups were not quite significant to reduce the bacterial loads of the dibiterie meat compare to the control group.

### Costs of hygiene improvement and intervention package, and its impact on the economic outcomes of dibiteries

The cost of hygiene improvement and the daily cost of the hygiene kit were low and estimated to average around 41 FCFA (USD 0.07) and 6 FCFA (USD 0.01) per day respectively (Table 5). The cost of the one-day training workshop was estimated at 440,000 FCFA (USD 789). Considering the 20 dibiteries that followed the training workshop and the fact that this intervention must be carried out once a year, the average cost of the training would amount to 22,000 FCFA (USD 39) per year per dibiterie, making 61 FCFA (USD 0.11) per day. Consequently, the cost of the intervention package (training + hygiene kit) was estimated at 67 FCFA (USD 0.12) per day per dibiterie.

**Table 5:** Estimation of the cost of hygiene and interventions package, and economic outcomes of dibiteries in Dakar

Rubric	Without intervention (i)	With intervention (f)
	Amount (FCFA)	Amount (FCFA)
1. Cost of equipment	13,219	13,358
2. Cost of consumables	3373	4470
3. Total cost of equipment and consumables (1 + 2)	16,592	17,827
Cost of hygiene (FCFA/month)	1236 (~ 41 FCFA/jour)	
4. Own capital	111,731	112;832
5. Loans	0	0
6. Fixed assets	2612	2616
7. Revolving funds [4 – (5 + 6)]	109,119	110,216
8. Investment on equipment and material	469,515	474,515
9. Total of revolving funds and investment (7 + 8)	578,634	584,730
10. D_x_ (day)	990	
11. Cost of hygiene kit (FCFA/day)	6	
12. Cost of the training (FCFA/day)	0	61
Cost of intervention package (training + hygiene kit) (FCFA/day)	67	
Revenue figure (FCFA/day)	122,505	122,505
Total variable charges (FCFA/day)	108,415	109,511
Gross margin (FCFA/day)	14,090^a^	12,994^a^

^a^ values followed by the same letter on the same row are not significantly different (t-test, *p* > 0.05); D_x_: the life period or use of the equipment invested (day)

The financial appraisal shows that dibiteries generate a daily gross margin of 14,090 FCFA (USD 25) without intervention and 12,994 FCFA (USD 23) with intervention; but no significant difference was noted between these two values. Thus, 1096 FCFA (USD 1.99) were lost per day with the intervention and the overall losses due to the introduction of the intervention are estimated to 1163 FCFA (USD 2.11) per day, representing 8.25% of the gross margin.

### Incentive factors to sustainably enhance good hygiene practices

#### Dibiterie tenants’ attitudes regarding the regulations surrounding hygiene and production of foodstuffs

Prior to undertaking the activity of processing and selling braised meat, the dibiterie tenant must be in possession of a medical certificate proving a clean bill of health, immunization against target communicable diseases which requires regular examination at a frequency determined by a sworn health service. Overall, dibiterie tenants are aware of the obligation to know their health status in order to exercise or carry out food production activities. A participant during the focus group discussion (FGD) states:

“*Yes, having a medical certificate is an obligation. We go to the hygiene service to make a medical examination and be able to take the medical certificate. This visit is done every 3 months. Without this paper, the hygiene and veterinary service officers will not allow you to sell dibiterie meat*” (FGD with a dibiterie tenant, Dakar, March 2018).

However, it also emerged that, in the opinion of some dibiterie tenants interviewed, that this regulation is not followed everywhere:

“*… this issue of medical examination, it is not all the people who respect it. There are some who do it and others not, because they operate clandestinely…*” (FGD with a dibiterie tenant, Dakar, March 2018).

Moreover, the dibiterie tenants are aware of the need to stop work when certain symptoms of diseases appear during handling of the meat, but rather than taking sick days, they seek treatment instead prior to continuing work. This is illustrated by the following statements:

“*… there is cough or tuberculosis, malaria, diarrhoea, stomach ache, vomiting, cold or fever. If I am working and these symptoms appear, the first thing to do is to get treatment before I come to continue working. I have to think about myself first, because I am sick*” (FGD with a dibiterie tenant, Dakar, March 2018).

“*… you have to stop and take the medicine. If you are two the second takes your place and you cure yourself. This is why the hygiene service officers ask for a pharmacy box*” (FGD with a dibiterie tenant, Dakar, March 2018).

#### Constraints related to the premise’s ownership

The lack of ownership and access to long-term rented premises represents an obstacle for sustainable investment in good hygiene practices in the dibiteries. According to dibiterie tenants, certain investments in infrastructure and hygiene equipment are only feasible when they own the workspace or the building or have a long-term user right. A participant during the focus group discussion stated:

“*On leasing, if it's not your building, there are certain facilities and expenses that you can't do, since the premises is not yours. If you do it, and the owner sees, he increases the rent*” (FGD with a dibiterie tenant, Dakar, March 2018).

The separation of clean and soiled sections of the premises is sometimes not carried out in some dibiteries and this fact, sometimes leads to inconvenience, frustration, discontent with customers. According to one of the participants, the need to achieve this separation is linked to the necessity to maintain customer privacy, and to maintain a secret proprietary recipe for braised meat produced in the dibiterie, as evidenced by the following statement:

“*Where I work, there is only one production room and I cannot put up partitions, because I am renting. But we need to keep the meat production process secret; and sometimes the smoke bothers customers, so you have to separate the eating room from the kitchen with a curtain... Also, for the privacy of customers, because there are some who come to eat discreetly, because it is sometime badly perceived by society. Other people also come to spend time with friends. But when you rent the premises for example, you can't concretely do separation, and the smoke makes customers' clothes smell and they complain*” (FGD with a dibiterie tenant, Dakar, March 2018).

#### Dibiterie tenants’ perceptions toward personal hygiene

The dibiterie tenants have a poor perception level regarding the use of regulation clothing. According to them, wearing regulation clothing, such as white coat, cap, apron is not synonymous with producing good quality meat. It’s just a marketing strategy among customers. This is illustrated by the following statement:

“*Wearing the white coat, cap and apron only attracts customers because the white colour represents cleanliness and they therefore find you more hygienic. When you go to a dibiterie where people are in white it attracts customers, but in fast food restaurants, for example, people wear Lacoste’s which are not necessarily white. What's important here is to wear something clean*” (FGD with a dibiterie tenant, Dakar, March 2018).

In the dibiterie, the use of metallic cut resistant/chainmail or disposable gloves, as well as the systematic washing of hands after each interruption, such as processing a sale or handling cash when handling meat are not carried out, due to financial and time constraints. This is illustrated by the statements of a dibiterie tenant during the focus group discussion:

“*Metal gloves are too expensive (~ 125 euros per pair), this is why I don't use them. Regarding the disposable gloves, I don't use them also, because it's veterinary officers who need them at the slaughterhouse*. *Washing hands is very important because of hygiene. But we can't always wash our hands at every interruption when customers are waiting. It's a matter of time*” (FGD with a dibiterie tenant, Dakar, March 2018).

#### Attitudes of customers during purchasing of dibiterie braised meat

The attitudes of customers purchasing braised meat seem to guide the implementation of good hygiene and production practices in the dibiteries. The recycled cement bag paper used for wrapping braised meat is the one preferred by customers due to the improved organoleptic quality of the braised meat. Participants during the focus group discussion declared:

“*There are customers who come to pay to take away and if you don't have any recycled cement bag paper it's useless, it's the job that requires it. Sometimes cement bag paper is missing, so we are obliged to get recycled milk bag papers*” (FGD with a dibiterie tenant, Dakar, March 2018)

“*I use the recycled cement bag papers because that's what customers ask for. Some people ask that the meat be braised in the cement bag paper, so that it is more succulent, more tender and has more taste; in addition, the meat does not adhere to this paper*” (GD with a dibiterie tenant, Dakar, March 2018).

The level of meat cooking is also chosen by the customer. However, according to dibiterie tenants, experience is the first quality criterion in the practice of the business of dibiterie braised meat processing and selling. It is instinct and experience that allows one to know if the meat is properly cooked according to the customer’s desire as evidenced by these statements:

“*... It depends on the customer. Sometimes there are people who like bloody meat, others don't. This is based on how we cook the meat. There are three ways to grill: bloody, cooked ‘a point’, and well cooked. It depends on the customer's request*” (FGD with a dibiterie tenant, Dakar, March 2018).

“*We know there is a thermometer that can tell if the meat is well cooked or not. But we don't use it because it's the experience that counts here. We know very well, just by looking at whether the meat is well cooked. If you know your job, you know whether the meat is good or not, just with from feeling or by a simple observation*” (FGD with a dibiterie tenant, Dakar, March 2018).

#### Dibiterie tenants’ perceptions toward the risks of foodborne diseases

Dibiterie tenants are aware that poorly stored and undercooked meat could contain bacteria that can spoil the food and cause illness among consumers, as evidenced by this statement from one of the participants:

“*... yes of course, often this is a meat conservation issue. I myself have been a victim. I once ate a Shawarma at a fast-food restaurant, I almost died that night. At 3 a.m. I was taken to the hospital, because the conservation was not good*” (FGD with a dibiterie tenant, Dakar, March 2018).

In addition, dibiterie tenants perceive that the risk of infection from their products is low. According to them, while this phenomenon may be recurrent in fast food restaurants, it is on the other hand rare in dibiteries because the meat is well cooked prior to consumption. However, the occurrence of foodborne diseases is sometimes linked to poor eating habits of consumers who mix foods up after consuming dibiterie braised meat. This is illustrated in the following statements:

“*... it's not the same thing in fast food restaurants and dibiteries. In the dibiteries, the meat is well prepared and cooked, so it is difficult for you to get a food poisoning. Even if there are unsold ones, the next day when you eat it can't give you stomach aches*” (FGD with a dibiterie tenant, Dakar, March 2018).

“*... often there are customers who mix things up. When they finish eating, they take sour milk and it gives them a food infection and then they think it is the dibiterie meat that is the cause. The ideal thing is to drink something hot, such as ‘Kinkeliba’, coffee, or hot water plus lemon*” (FGD with a dibiterie tenant, Dakar, March 2018).

From the opinions of the participants, it emerged that belief in God is important in providing quality meat free from any microbial contamination. However, they are aware that to avoid foodborne diseases for consumers, it is necessary to ensure proper storage and cooking of products, as evidenced by the following statement:

“*When you believe in God and sell braised meat, there will be no more bacteria in dibiterie braised meat. Because everything that is put on fire is safe and you can consume it without worry. But care must be taken to ensure that the meat is safely stored and well-cooked beforehand*” (FGD with a dibiterie tenant, Dakar, March 2018).

## Discussion

The present randomized controlled trial study aimed at designing an incentive-based health intervention to improve the hygiene and safety of dibiterie meat, and the health of consumers in Dakar. The study format was chosen because intervention studies without control group can suggest that the positive changes may be linked to other factors [13]. In addition, we have taken care to randomly distribute the different intervention groups so that they cannot interact with each other, and to be able to better appreciate the effectiveness of the innovations and messages promoted as part of the intervention.

### Hygiene behaviour in the dibiterie

In Senegal, practicing any collective commercial catering activity requires the presentation of a certificate of good health, in accordance with the hygiene code (Law No 83.71 of July 5, 1983) [14]. Most of the dibiterie tenants (70%) did not respect this principle prior to commencing trading, which indicates poor attitudes and hygiene practices in line with their position as an informal business. This behaviour is linked to their strategy of minimizing costs which would also lead them to avoid any census or registration in order to avoid paying taxes like any legally registered business must. The same observations were made by Gitahi [15] in East Africa, where 76% of Kenyan informal vendors did not have a medical certificate to practice. In contrast, Annan-Prah et al. [16] and Ackah et al. [17] in West Africa found that 55–60% of food vendors in the informal sector in Ghana had a medical certificate to handle food. These studies seem to reveal that in Africa and particularly in the informal sector, many traders operate without respecting the principle of good health. This situation is linked to the lack of strict control and monitoring of good hygiene practice rules in the informal sector. In catering, the possession of a medical certificate is of utmost importance to ensure that people who handle food are immunized or treated against typhoid and other foodborne diseases to avoid cross-contamination [18].

Moreover, the transport of meat by non-refrigerated means, and the exposed meat to open air are unhygienic practices which expose the meat to microbial contamination of exogenous or environmental origin. Indeed, these practices lead to a break in the cold chain conducive to the multiplication of pathogenic bacteria following contact of the meat with insects, ambient air, and aerosols produced by mobile devices [19]. According to AFSSA [20], refrigeration limits the activity or growth of bacteria likely to contaminate food and any hazard is avoided by keeping the meat at a temperature below or equal to 5 °C.

Regarding personal and clothing hygiene, the majority of dibiteries sampled as part of the intervention did not comply with basic good hygiene practice measures. Handling money, wearing jewellery by the seller or the butcher, and not washing hands after each interruption during the production process are unhygienic behaviours that would promote bacterial contamination of the meat. This lack of knowledge regarding the basic rules of good hygiene practices could be linked to the low level of formal training of the dibiterie tenants. The same observations were made by Baba-Moussa et al. [19] in Benin where the majority of street food vendors with low levels of education handle food by hand. According to the authors, the lack of education results in ignorance of the basic rules of hygiene and safety. Indeed, hands and money both coins and notes often serve as vectors of microbial contamination of foodstuffs and may be the cause of foodborne diseases (diarrhoea, gastroenteritis) among consumers [19, 21, 22]. The hands play an important role in the contamination and spread of foodborne bacteria, and the risk increases when sellers use their bare hands during handling and sale [21].

### Sources of microbial contamination of dibiterie meat

The results of this study showed that in phase 1 (one-month pre-intervention), the dibiterie meat was contaminated with bacteria such as TAMF, TC and SA. In fact, 27 and 13% of the samples respectively contained TC and SA exceeding international standards. On the other side, no sample contained TAMF exceeding microbiological quality standards in phase 1. The presence of these bacteria suggests poor hygiene and production practices of dibiterie tenants. This deficit, according to Barro et al. [21], Mensah et al. [23] and Bryan et al. [24], are possible means of transmission of pathogens in food. In addition, Pesewu et al. [25] and Amponsah-Doku et al. [26] indicated that the persistence and substantial proliferation of bacteria from food production / processing to consumption reflects poor sanitary and food handling practices, as well as conditions at various stages of the storage chain. TAMF consists of poor hygiene indicative bacteria attesting to a failure at the level of overall hygiene. Since the load of TAMF in the dibiterie meat samples does not exceed the quality standards one-month pre-intervention, this means that the overall hygiene of the dibiterie, that is to say the cleaning and disinfection procedures carried out therein, are therefore acceptable. However, the presence of TAMF bacteria in dibiterie meat samples despite cleaning and disinfection operations could be related to the lack of separation of cleaning and disinfection procedures leading to ineffective disinfection. Indeed, during the cleaning and disinfection of small kitchen utensils (knives, machete, containers), the water used is often not renewed or changed for all the small utensils, which are subsequently exposed to the open air without any coverage. Regarding TC and SA, their presence testifies the contamination of dibiterie meat after cooking. The relatively high percentage of non-satisfactory samples could be mainly explained by (i) the defective personal and clothing hygiene of the staff who, moreover, are considered to be the main source of contamination, (ii) the lack of or poor use of sanitary facilities (defective maintenance, inadequate fittings, lack of soap and toilet paper), and (iii) incorrect and irregular hand washing. The study also showed that 85% of dibiterie tenants did not wash their hands after each interruption during the production process. Post-cooking contamination could also come from raw ingredients or spices added to meat after cooking, thus leading to cross-contamination [27, 28].

### Effectiveness and cost of the intervention

The present study has shown that the microbiological quality of dibiterie meat can be improved through simple, practical interventions based on participant incentivization. Indeed, even if the differences were not statistically significant, the various interventions implemented still showed decreases in the sample proportions of non-satisfactory quality for each of the microbiological indicators tested in phase 2. On the other hand, in phase 3, the microbiological quality of the samples was improved significantly by 18.5% (*p* < 0.1) for the whole bacteria species analysed (TAMF, TC and SA) and by 19% for the TC (*p* < 0.1). The intervention, therefore, lead to a significant improvement in the general hygiene of the dibiteries, the personal hygiene of the staff, and reduced post-cooking contamination of the meat. Thus, in phase 3, the dibiterie meat samples are 4.4 and 3.3 times less likely to be of non-satisfactory quality for TC, and for the whole bacterial species range analysed (TAMF, TC and SA) respectively.

The study also showed that training is the most effective intervention to reduce the bacterial load in dibiterie meat. The effectiveness of the hygiene kit, and the training + hygiene kit was not statistically significant. The fact that the training has been effective alone but not when combined with the hygiene kit could suggest that incentives to apply the good hygiene practices may have decreased with the depletion of the hygiene kit received by the dibiterie tenants during the intervention, who may have failed to replace these products. This means that the improvement of hygiene practices in the dibiterie is not affected by the variable factors of production, such as materials and consumables for hygiene. Moreover, the training has significantly reduced the total bacterial load in phase 3 but not in phase 2. Therefore, the training did not significantly improve the overall hygiene and especially the personal hygiene at the phase 2 because it is very difficult to suddenly change traditional behaviours which are often very rooted in habits [8, 29, 30]. Indeed, phase 2 allowed the dibiteries to gain experience implementing the messages of the training intervention. This time was therefore not sufficient for the effective adoption of the messages and innovations promoted. In contrast, the messages and innovations promoted during the training were found to have been adopted 10 months later post-intervention. This indicates that this is the time it took for dibiterie tenants to fully implement the messages and innovations in their establishments. Therefore, refreshment courses are needed at least once a year for sustainable behaviour changes.

Improvements in hygiene and food safety in the informal system are therefore possible even when actors lack formal education and adequate production infrastructure. Several studies have shown the effectiveness of hygiene education interventions for food processing actors operating in different food sectors [11, 13, 31–34].

The daily cost of the intervention package is low, and estimated on average at USD 0.12 per day, i.e., USD 0.1 per day for training, and USD 0.01 per day for the hygiene kit. In addition, USD 1.99 were lost per day with the intervention implementation and the overall losses represents 8.25% of the gross margin due to the introduction of the hygiene intervention. However, the assessment of the intervention costs has not account for possible health benefits of consumers not becoming ill, and of possibly preventing some foodborne illness. Moreover, these costs could be well supported in the production cost of the dibiteries given the strong economic viability and profitability of these establishments. This quality production could therefore allow an increase in the selling price of braised meat. Indeed, a recent study had shown that 84% of dibiterie meat consumers are ready to pay up to USD 0.84 more on the purchase prices to improve the defects of the dibiterie meat quality [35]. In addition, this price increase associated with the scale economy strategy (increasing returns to scale) could help to absorb these losses [36].

Moreover, no significant difference was observed between the gross margin of the dibiterie with and without the intervention. The cost of hygiene estimated at USD 0.07 per day, therefore, does not influence the economic gain of the dibiteries.

The cost of the intervention hygiene kit obtained in the present study is lower than that obtained by Bonfoh et al. [11] in the dairy value chain in Mali estimated on average at USD 0.13 per day. This difference in costs observed could be linked to the fact that improving hygiene in dairies in Mali requires more investment than that of dibiteries in Senegal. Moreover, the cost of training butchers at the slaughterhouse was estimated on average at USD 9 per butcher in Nigeria, and generated savings estimated at USD 780 [13].

Finally, the intervention may have worked but the results are mixed and not quite significant. Indeed, there are some borderline significant reductions in bacterial contamination at phase 3 for the 2nd intervention arm (group b); but overall, there are (i) no effectiveness at phase 2, whereas normally intervention effect must be highest early on, (ii) no benefit with the 4th intervention arm (group d), which is the association of the 2nd and 3rd intervention arms. Consequently, we recommend further intervention refinement and testing.

### Incentive and motivation factors for sustainable hygiene practices in dibiteries

Hygiene and quality management in dibiterie restaurants are affected by the behaviour and perception of dibiterie tenants, the purchasing practices of customers, and mainly economic and institutional factors.

In terms of behaviour and perceptions, the dibiterie tenants are aware of the hazards and risks associated with poor hygiene practices on food safety and the health of consumers. However, their behaviours are sometimes culturally rooted and income, convenience dependant. Indeed, the dibiteries have a good knowledge of the techniques and production processes for preparation of braised meat, the quality, and the associated risks, but their attitudes differ greatly from their actual hygiene practices. Evidence on the risks and the result of focus group discussion showed that the theoretical statements of the dibiterie tenants are not always realised in practice. This is due to the fact that the practice and the business of producing and selling braised meat in dibiteries is done from an early age for some and it continues from generation to generation [36]. Thus, the training acquired from parents, friends, or other members of the family remains culturally rooted in habits and becomes very difficult to change. The same remarks were made by Mahamat [37] in the dairy sector in Niger where personal and clothing hygiene were difficult to improve due to the traditional habits of the staff. Furthermore, the non-demanding from clients does not tend to enforce application of hygiene and the production of good quality meat. Indeed, consumer purchasing decisions are not always guided by meat safety factors. The perception of quality among dibiterie meat consumers is oriented towards organoleptic or sensory quality (taste, flavour, tenderness, etc.). Orou Seko et al. [35] found that 61% of consumers were less concerned about health factors when buying braised meat in dibiteries.

Economic and institutional factors also affect the management of hygiene and food safety in the dibiteries. Indeed, certain economic factors such as human resources, the ownership risk linked to the lack of owner status of the premises, financial, and time constraints limit the application of the conventional good hygiene practice rules promoted herein in the dibiteries. Basic good hygiene practices such as handwashing and wearing gloves are not always carried out by the dibiterie tenants due to the lack of human resources, time, and financial means. These constraints lead to difficulties in organisation, distribution of tasks, and investment in sustainable infrastructures for quality production within the dibiteries. Therefore, the meat is exposed to possible cross-contamination which may arise from the staff and the environment within the dibiterie premises. Although the current dibiterie business model is highly profitable, it makes hygiene and public health interventions difficult to implement, because of their precarious work setting, which limits sustainable investments in the infrastructures by the dibiterie owners. Thus, the main incentive for the sustainable adoption of good hygiene practices by dibiteries was access to sustainable and secure infrastructure.

**Fig. 3:**
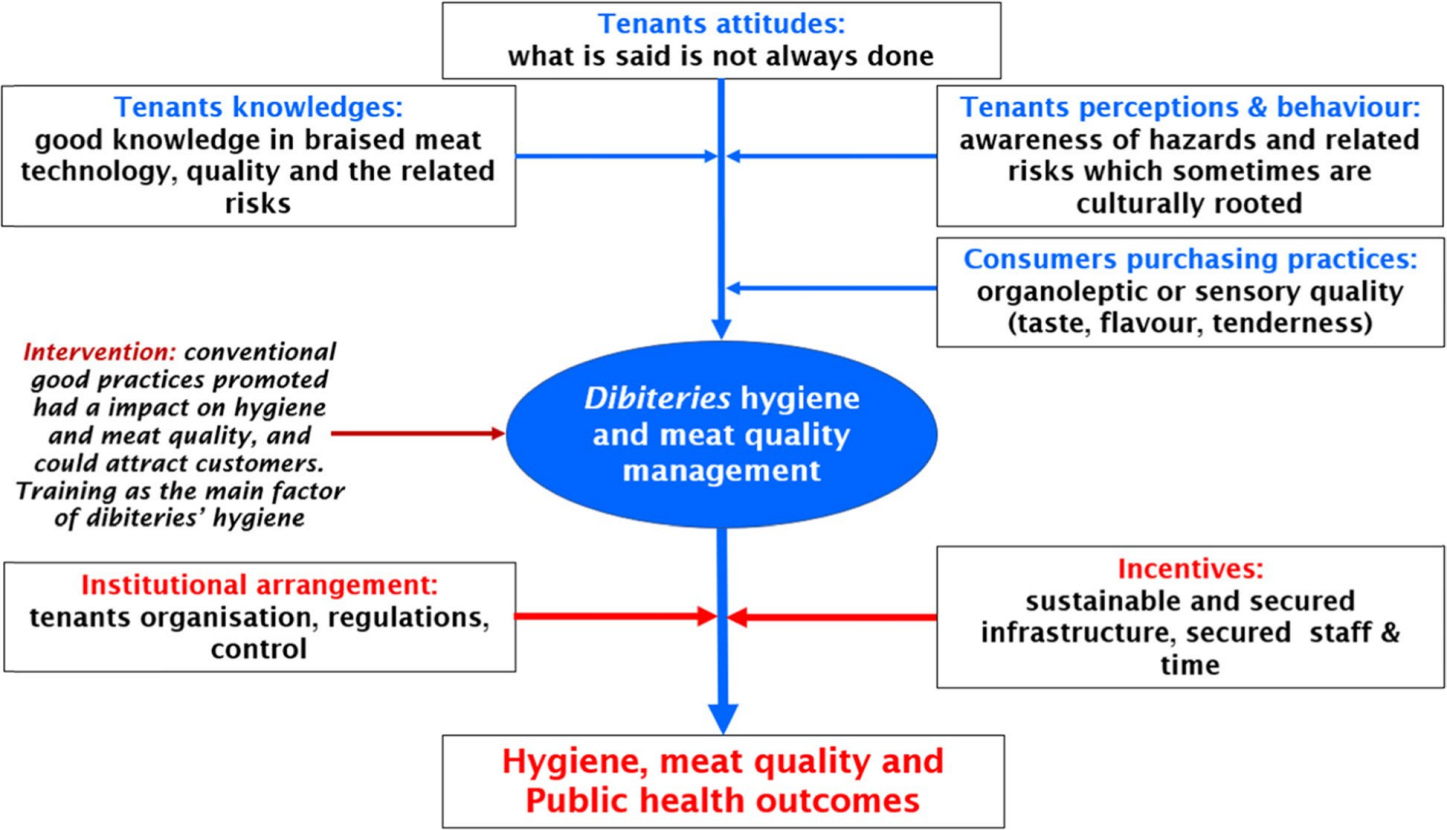
Dibiteries hygiene and meat quality management framework and public health implications

Participatory design of adapted quality standards and organisation of the dibiteries through a system to access a secure tenure spaces in urban setting by the public institutions (municipalities, prefectures, etc.) could boost investments, promote access to credits as well as trainings on good hygiene practices for dibiteries. Improving hygiene management and meat quality in the dibiteries must therefore take into account food hygiene behaviour of the dibiterie tenants, consumers’ purchasing practices. Finally, the incentivization of the dibiterie tenants relating to institutional and financial arrangements is necessary to enhance the sustainability of hygiene management practices in the dibiteries. Figure 3 shows the dibiterie hygiene and meat quality management framework and public health implications considering the socio-economic and environmental determinants of hygiene.

### Limitations of the study

Firstly, the intervention strategies were based on the sample of 40 dibiteries previously evaluated by Yougbare [7]. The small sample size per group, could be linked to the relatively low statistical power. Secondly, although the tenants in the four intervention groups had very similar descriptive characteristics, the intervention strategies did not take into account the technological specifications of braised meat production according the type of dibiterie. The microbiological analyses were conducted on the combined braised meat and ingredients to better evaluate the overall importance of the intervention strategy. Future research should be conducted with a larger number of dibiteries and separate analyses of braised meat and ingredients. Thirdly, the meat contamination bacterial indicators reflect only a point in time estimate of hygiene, and they may not reflect the overall food safety improvements of the facility. Additionally, if the food handlers were aware they were preparing the specific meat samples for testing, they may have altered their normal practices for this purpose, which could have affected the results. These considerations raise hypothesis which could be tested in the future research, especially (i) looking at changes in food safety behaviors pre- and post intervention across the groups in addition to the bacterial indicators; (ii) identifying the intervention improvements when evaluating food handler behaviors versus meat contamination.

## Conclusion

The microbiological quality of dibiterie meat can be improved through well co-designed and adapted interventions to the informal sector which very often escapes safety control. Although the cost of the intervention package is low, in overall the results of the intervention were insufficient, which suggesting the need for more aggressive / expansive intervention. However, the hygiene training was more effective in reducing the microbial load and generates quality and long-term financial benefits. In addition, the implementation of the intervention does not significantly impact the production revenue of the dibiteries, and therefore is not the limiting factor in adopting innovations. Access to sustainable and secured infrastructure space was the main incentive for the sustainable adoption of good hygiene practices by dibiterie tenants. For a sustainable change in the hygiene behaviour of the dibiterie, hygiene education interventions should be combined with the promotion of access to secure workspaces for dibiterie owners. Finally, institutional arrangements such as participatory health regulation and sanitary control as well as promoting investment in infrastructures will enhance the health of consumers.

## Data Availability

The datasets used and/or analysed during the current study are available from the corresponding author on reasonable request.

## Abbreviations

CFU: Colony Forming Unit
SA: *Staphylococcus aureus*
TAMF: Total Aerobic Mesophilic Flora
TC: Thermotolerant Coliform
WHO: Word Health Organisation
